# Development and Validation of a Rapid High-Performance Liquid Chromatography–Tandem Mass Spectrometric Method for Determination of Folic Acid in Human Plasma

**DOI:** 10.3390/ph11020052

**Published:** 2018-05-27

**Authors:** Aref Zayed, Rana Bustami, Wafaa Alabsi, Tamam El-Elimat

**Affiliations:** 1 Department of Medicinal Chemistry and Pharmacognosy, Faculty of Pharmacy, Jordan University of Science and Technology, Irbid 22110, Jordan; telimat@just.edu.jo; 2 Pharmaceutical Research Unit, Amman 11910, Jordan; rtbustami@pru.com.jo (R.B.); wafaa_alabsi@yahoo.com (W.A.)

**Keywords:** high-performance liquid chromatography–tandem mass spectrometry, electrospray ionisation, folic acid, validation, human plasma

## Abstract

There are health concerns associated with increased folic acid intake from fortified food and supplements. Existing analytical methods, however, which can be employed to carry out epidemiological and bioavailability studies for folic acid involve laborious sample preparation and/or lengthy chromatographic analysis. In this paper we describe a simple, rapid, and sensitive high-performance liquid chromatography–electrospray ionisation-tandem mass spectrometry (HPLC–ESI-MS/MS) method for determination of unmetabolised folic acid in human plasma using folic acid-d4 as an internal standard. The method required only a simple sample preparation step of protein precipitation and had a total run time of 3.5 min, which is the shortest run time reported to date for HPLC–MS/MS method employed for quantifying folic acid in plasma. The analytes were separated on a C18 column (3 µm; 50 × 3.00 mm) using an isocratic mobile phase consisting of ammonium acetate (1 mM)-acetic acid-acetonitrile (9.9:0.1:90, *v*/*v*/*v*). The method was fully validated in terms of accuracy, precision, linearity, selectivity, recovery, matrix effect, and stability. The short run time and the minimal sample preparation makes the method a valuable tool for performing high-throughput analyses. To demonstrate the applicability of the method in real conditions, it was applied successfully in a bioavailability study for the determination of unmetabolised folic acid levels *in vivo* in human plasma after oral administration of folic acid.

## 1. Introduction

Folate is a water-soluble B vitamin that is also known as vitamin B_9_ or folic acid [1]. It is a generic term referring to a group of structurally related compounds that have the same basic skeleton but differ in the oxidation state and the number of glutamate moieties [2,3]. Naturally occurring folate exists in food in polyglutamate reduced forms [3]. The two principal reduced forms are 7,8-dihydrofolate (DHF) and 5,6,7,8-tetrahydrofolate (THF) [2]. Folic acid is a synthetic monoglutamate oxidised folate with remarkable stability and bioavailability that makes it the folate form of choice for supplementation and fortification of food products [2,3,4]. To exert biological activity, folic acid should be reduced to the metabolically active tetrahydrofolate form which mainly takes place in the intestine mucosa and liver [5]. The synthetic folic acid vitamin, however, can appear in the circulation after passing unchanged from the intestine following ingesting a high dose of oral supplements or fortified food [6].

Folate coenzymes function as a single-carbon donor and acceptor in a number of critical biochemical reactions for DNA and amino acid metabolism [3]. Folate deficiency is implicated in the pathogenesis of a number of diseases, including megaloblastic anemia [7,8], cardiovascular disease [9,10], colorectal cancer [11,12], and cognitive dysfunctions [13,14]. During pregnancy, impaired folate status has been associated with abnormal pregnancy outcomes, such as increased incidence of habitual spontaneous abortion, low birth weight, preterm delivery [15,16], and increased risk of birth defects, such as congenital heart defects [17] and neural tube defects (NTDs) [18,19]. To prevent the incidence of NTDs [20] and other related folate deficiency defects, many countries have implemented a mandatory fortification of flour and cereal products with folic acid [21].

Excess folate consumption from food is considered safe. There are, however, safety concerns associated with excessive doses of folic acid [4]. Tolerable upper intake levels (ULs) of folic acid should not exceed 1 mg/day [4]. Folic acid is an oxidised form of folate that should be metabolised in the liver into tetrahydrofolate (active form) by tetrahydrofolate reductase (THFR) [3]. Many reports have suggested that this enzymatic conversion is slow [22]. Moreover, THFR has limited capacity and may eventually get saturated [23], resulting in detrimental increased level of unmetabolised folic acid in plasma or tissues [24,25,26,27]. Large doses of folic acid may mask vitamin B_12_ deficiency, leading to irreversible neurological damage [28,29]. A decreased activity in natural killer cells was reported in elderly women with high plasma levels of unmetabolised folic acid [29]. High folic acid intake might also aggravate pre-existing cancers or accelerate the progression of preneoplastic lesions, increasing the risk of colorectal and prostate cancers, for example [30,31,32]. Thus, it is very important to develop selective, sensitive, and accurate analytical methods for determination of unmetabolised folic acid in plasma.

Different approaches were developed for folate analysis in biological systems. Microbiological assay [33,34] was the first method introduced, followed by protein-binding assay [34,35], and finally chromatographic methods [35]. The microbiological assay is a simple, inexpensive, and sensitive method. It is, however, an imprecise and time consuming assay that has been surpassed by the protein-binding assay [35]. A protein-binding assay is a simple, high-throughput, and cost-effective method, but with large interassay variations depending on the commercial kit used [34,35]. Both methods are used for total folate measurements in blood samples [35]. Hence, chromatographic assays were introduced as more specific and selective methods [34]. A number of high-performance liquid chromatographic (HPLC) methods were reported in the literature for quantification of folates in human plasma, such as HPLC–UV [36], HPLC–FLD [37], HPLC–ECD [38], HPLC–MS [39], and HPLC–MS/MS [40]. However, none of these methods were convenient for routine and high-throughput analysis due to several reasons, including laborious sample preparation, such as solid-phase extraction [41,42], and lengthy chromatographic methods, such as hydrophilic interaction chromatography [43] or gradient reversed-phase chromatography [40]. Evidently, tandem mass spectrometry (MS/MS) methods were the best with enhanced sensitivity, selectivity, and specificity.

**Figure 1:**
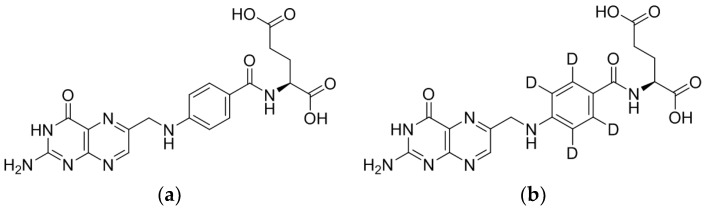
Structures of (**a**) folic acid and (**b**) folic acid-d4 (internal standard).

In this study, a 3.5-min isocratic HPLC–MS/MS method with a quick sample preparation, requiring only acetonitrile protein precipitation, was developed and validated for quantification of unmetabolised folic in human plasma using folic acid-d4 as an internal standard (Figure 1). To the best of our knowledge, this HPLC–MS/MS method has the shortest runtime reported for the quantification of folic acid. Combined with a simple and rapid sample preparation, it is an ideal method for carrying out studies requiring high-throughput routine analyses such as in bioavailability and epidemiological studies.

## 2. Materials and Methods

### 2.1. Materials and Reagents

Reference standards of folic acid (98.6%) and folic acid-d4 (98.0%) were purchased from Toronto Research Chemicals (Toronto, ON, Canada). HPLC-grade methanol and acetonitrile were obtained from ROMIL Ltd. (Cambridge, UK). Ammonium acetate and acetic acid, both of ACS reagent grades, were obtained from Merck KGaA (Darmstadt, Germany). Pure water was prepared using the Sartorius water purification system (Sartorius, Goettingen, Germany).

### 2.2. Liquid Chromatography Tandem Mass Spectrometry

The chromatography system used was an Agilent 1260 Infinity HPLC system, which was equipped with quaternary pump, standard autosampler, and thermostatted column compartment from Agilent Technologies, Inc. (Santa Clara, California, CA, USA). Chromatographic separations were achieved using a Luna C18 HPLC column (3 µm; 50 × 3.00 mm) with a C18 guard column, both from Phenomenex Inc. (Torrance, CA, USA). Column oven temperature was set at 25 °C. An isocratic mobile phase was used, which consisted of ammonium acetate (1 mM)-acetic acid-acetonitrile (9.9:0.1:90, *v*/*v*/*v*) (pH 3.4). The flow rate used was 0.5 mL/min and the injection volume was 20 µL.

**Figure 2:**
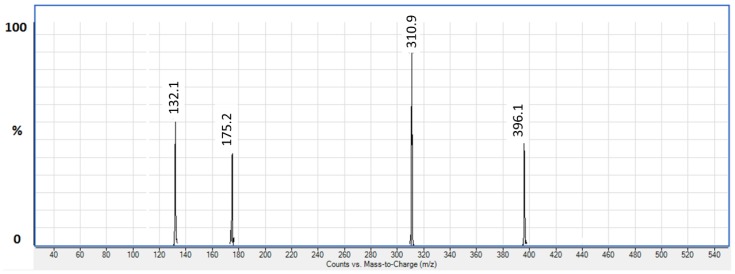
Product ion mass spectrum of folic acid (*m*/*z* 440.40 → 310.90) in the negative ionisation.

Folic acid and folic acid-d4 were detected using an Agilent 6490 Triple Quadrupole tandem mass spectrometer (Agilent Technologies, Inc., Santa Clara, CA, USA), equipped with electrospray ionisation (ESI) operating in negative ionisation mode. Folic acid and folic acid-d4 were monitored in multiple reaction monitoring (MRM) mode. The following MRM transitions, *m*/*z* 440.40 → 311.10 and *m*/*z* 444.40 → 315.00, were monitored for folic acid and folic acid-d4, respectively, with collision energy set at 21 eV. Determination of compound MRM parameters, including fragmentor voltage and collision energy were done automatically by the LC–MS/MS software (Agilent MassHunter^®^ Workstation ver B.06.00, Agilent Technologies, Santa Clara, CA, USA), ensuring reproducibility of the assigned parameters. The same values were obtained on repeated tuning experiments. Figure 2 shows the typical product ion scan spectrum of folic acid. The MS source parameters were as follows: nebuliser gas of 50 psi, sheath gas flow of 11 L/min at a temperature of 290 °C, and capillary voltage of 3000 V. Agilent Mass Hunter Software Version B.06.00 was used to control all parameters of HPLC and MS.

### 2.3. Preparation of Standard Stock, Calibration Standards, and Quality Control Samples

Standard stock solutions of folic acid (725.7 µg/mL) and folic acid-d4 as an I.S. (19.60 µg/mL) were prepared by dissolving 40.0 mg folic acid (assay 98.6%, loss on drying 92.0%) and 1.0 mg folic acid-d4 (assay 98.0%), respectively, in 50 mL of 0.1% ammonium hydroxide-methanol (40:60, *v*/*v*) . Additionally, a working stock solution of folic acid (72.57 µg/mL) was prepared by diluting 1.00 mL from the former folic acid stock solution (725.7 µg/mL) to a final volume of 10 mL using methanol. Calibration curve standards (CCs), quality control (QC), and dilution integrity evaluation (DIE) samples were prepared by spiking human blank plasma with pre-prepared working solutions of folic acid stock solutions. The following calibration curve points were prepared: 13.17 ng/mL, 26.33 ng/mL, 131.7 ng/mL, 439.9 ng/mL, 1024 ng/mL, 2194 ng/mL, 3292 ng/mL, and 3657 ng/mL. The QC samples were prepared at five concentration levels: 13.17 ng/mL (lower limit of quantification (LLOQ)), 39.50 ng/mL (low QC (QCL)), 731.5 ng/mL (medium QC (QCM-1)), 1829 ng/mL (medium QC (QCM-2)), and 2743 (high QC (QCH)). For DIE, the following concentration levels were prepared: 72,576 and 10,885 ng/mL. Plasma samples were stored at −70 °C, while stock solutions were stored at −20 °C.

### 2.4. Sample Preparation

Prior to extraction, to an aliquot of 500 µL of spiked CCs, QC, and DIE samples, 50 µL of the I.S. stock solution (19.60 µg/mL) was added and vortexed for 30 s to obtain a final I.S. concentration of 1781 ng/mL. After that, about 1.5 mL of ice-cold acetonitrile was added to each sample followed by vortexing for 30 s. The samples were then centrifuged for 10 min at 14,000 rpm and 800 µL of the supernatant was decanted and evaporated under a stream of nitrogen gas at a temperature of 40 °C until complete dryness. The residue was then reconstituted with 200 µL of mobile phase and 20 µL was injected into the column for HPLC–MS/MS analysis.

### 2.5. Method Validation

The method was fully validated in accordance with Bioanalytical Method Validation guidelines issued by US Food and Drug Administration [44].

The linearity of the method was examined by preparing nine calibration curves with eight nonzero concentrations (13.16 ng/mL, 26.33 ng/mL, 131.7 ng/mL, 439.9 ng/mL, 1024 ng/mL, 2194 ng/mL, 3291 ng/mL, and 3657 ng/mL). Peak area ratios of folic acid/folic acid-d4 were plotted against folic acid concentration.

Within-run accuracy and precision (intrabatch precision or within-run repeatability) were assessed by measurement of folic acid in plasma samples at five concentration levels (LLOQ (13.17 ng/mL), QCL (39.50 ng/mL), QCM1 (731.5 ng/mL), QCM2 (1829 ng/mL), and QCH (2743 ng/mL)), each run in six replicates. Peak areas were calculated and analysed against a calibration curve that was prepared on the same day. Between-run accuracy and precision (interbatch precision or between-run repeatability) were determined by analysis of five separate accuracy and precision batches, each run in six replicates on three consecutive days. Peaks were calculated and analysed against a calibration curve that was prepared alongside each batch.

The extraction recovery of folic acid and folic acid-d4 from human plasma was examined in six replicates at four concentration levels—QCL, QCM-1, QCM-2, and QCH—by comparing the mean peak area ratio of folic acid/folic acid-d4 after extracting spiked samples with mean peak area ratio of folic acid/folic acid-d4 of samples prepared in mobile phase (100% recovery) with identical concentration.

The matrix effect of the method was evaluated by analysing six different lots of blank plasma spiked with folic acid at QCL and QCH concentrations after extraction, in comparison to pure solutions of folic acid. The matrix factor (MF) for folic acid and folic acid-d4 was calculated by estimating the area ratio of the peaks in the presence and absence of matrix. The MF was normalised by dividing the MF of folic acid by the MF of the internal standard.

Stability of folic acid was evaluated at QCL and QCH at different conditions, including short-term stability of spiked plasma samples at room temperature up to 24 h, short-term stability in blood for 1 h at 15–25 °C, freeze and thaw stability for four cycles at −20 °C and −70 °C, auto-sampler/dry phase at room temperature and −20 °C for about 125 h, auto-sampler/injecting phase at reference temperature and room temperature for 124 h, long-term stock solution stability over 9 days and short-term stock solution stability over 3 days at LLOQ and ULOQ, long-term stock solution stability over 10 days, and short-term stock solution stability over 3 days of IS. Folic acid/folic acid-d4 peak areas ratio of stability samples were compared with freshly prepared standards with identical concentration.

Dilution integrity of the method was evaluated by analysing six replicate samples that were prepared as spiked standards at two concentration levels, 7315 and 10,972 ng/mL, representing approximately 2× and 3× the concentration of the ULOQ (3657 ng/mL) after 2- and 3-fold dilution, respectively. A freshly prepared calibration curve was then used to calculate the accuracy and precision of the samples.

Carry-over was assessed using the following sequence of injections: double blank, double blank, zero blank, upper limit of quantification (ULOQ) calibration sample (3657 ng/mL), zero blank, and lower limit of quantification (LLOQ) calibration sample (13.17 ng/mL). Zero blank/LLOQ ratio was then assessed.

The analytical method should be able to differentiate folic acid and I.S. from endogenous components in the plasma or other components in the sample. Selectivity was tested using six individual sources of blank plasma, which were individually analysed and evaluated for interferences. The selectivity of the method was also studied by checking the possibility of interferences from commonly used medications by human volunteers. Stock solutions of paracetamol (31.97 µg/mL), diclofenac (1.859 µg/mL), ibuprofen (94.94 µg/mL), caffeine (20.12 µg/mL), acetylsalicylic acid (100.0 µg/mL), and ascorbic acid (3.85 µg/mL) were prepared by dissolving appropriate amounts in 25 mL methanol. Appropriate volumes were then spiked with QCL samples and analysed using freshly prepared calibration curve standards. QCL samples analysed against the same calibration curve were then compared for interferences by calculating the accuracy of the samples.

## 3. Results

An LC–MS/MS method was developed and validated according to FDA guidelines [44] for quantification of folic acid in human plasma using folic acid-d4 as an I.S.

### 3.1. Method Development

#### 3.1.1. LC/ESI-MS/MS

Initial method optimisation experiments were carried out on both positive and negative ionisation modes. As better S/N for both folic acid and folic acid-d4 was obtained in the negative mode, it was selected over the positive mode for further method development and validation.

A solution of folic acid at a concentration of 1.0 µg/mL was prepared in methanol-ammonium acetate (5 mM). The solution was infused into the electrospray ion source at a flow rate of 10 µL/min for MRM optimisation. The LC–MS/MS spectra of folic acid using collision-induced dissociation (CID) of molecular ion *m*/*z* 400.4 showed several fragment peaks *m*/*z* 396, 311, 175, and 132 (Figure 2). The most intense fragment ion due to the loss of glutamic acid residue (*m*/*z* 311.1) was selected for the monitoring and quantification of folic acid. CID of folic acid-d4 molecular ion at *m*/*z* 444 gave a similar fragmentation pattern with fragments occurring at *m*/*z* 315, 175, and 132. Ultimately, the following MRM transitions, *m*/*z* 400.4 to *m*/*z* 311.1 and *m*/*z* 444.0 to *m*/*z* 315.0, were monitored for quantification of folic acid and folic acid-d4, respectively.

#### 3.1.2. Optimisation of the Chromatographic Conditions

**Figure 3:**
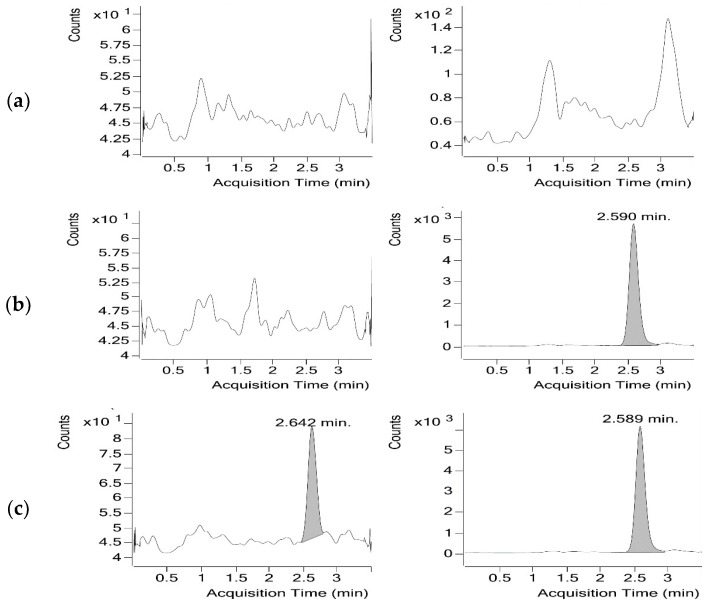
Multiple reaction monitoring (MRM) ion chromatograms of (**a**) A blank plasma sample. (**b**) A spiked plasma sample containing 1782 ng/mL folic acid-d4 (I.S.) tR = 2.590 min. (**c**) A spiked plasma sample containing 13.17 ng/mL folic acid (LLOQ) tR = 2.642 min and 1782 ng/mL folic acid-d4 (I.S.) tR = 2.589 min.

When optimising the chromatographic conditions for analysis of folic acid, the goal was to develop a fast and specific method that has the ability to measure accurately folic acid response in the presence of other plasma components, particularly potentially similar endogenous folate compounds. Various mobile phases’ composition and ratios, different buffers, and variable flow rates were tested over a C18 HPLC column (3 µm; 50 × 3.00 mm). An initial mobile phase was tested consisting of ammonium acetate (5 mM)-acetic acid-acetonitrile (9:1:90, *v*/*v*/*v*). Although an acceptable retention time of the drug and I.S. was obtained, this mobile phase was not used due to close elution of two small unknown peaks with the target compound. This problem seemed to be resolved by reducing the percentage of acetic acid and using a lower concentration of ammonium acetate. A mobile phase consisting of ammonium acetate (1 mM)-acetic acid-acetonitrile (9.5:0.5:90, *v*/*v*/*v*) resulted in better separation between folic acid and folic acid-d4 and the two unknown peaks. Moreover, the intensity of both folic acid and folic acid-d4 were doubled, potentially due to a better ionisation and reduced ion competition from buffer ions. Best results were achieved by reducing the concentration of acetic acid to 0.1% (*v*/*v*). Collectively, a fast and specific method was developed with short retention time and sharp peak shapes with optimal resolution (Figure 3) using an isocratic mobile phase consisting of ammonium acetate (1 mM)-acetic acid-acetonitrile (9.9:0.1:90, *v*/*v*/*v*) at a flow rate of 0.5 mL/min and an injection volume of 20 µL. The retention time for folic acid and folic acid-d4 were 2.64 and 2.59 min, respectively, and total run time was 3.5 min. Experiments with other mobile phase compositions by changing the buffer to ammonium formate and/or using methanol instead of acetonitrile did not result in enhancing the signal intensity or improving the chromatography.

For chromatographic methods, developing a separation involves demonstrating specificity, which is the ability of the method to accurately measure the analyte response in the presence of all potential sample components. One main advantage of the tandem mass spectrometry detector over other classical assays such as the microbiological assays and radio-immunoassays is its capability of high compound specificity particularly in the presence of similar endogenous folate compounds. The LC method proved to be selective and was able to separate folic acid even from compounds with similar masses and transitions. The chromatogram of blank plasma sample (Figure 3a) shows the elution of small amounts of two species (retention time ~1.3 and 3.2 min) of similar MS transition to folic acid-d4 (retention time 2.6). The difference in retention time demonstrates the selectivity of the separation and reliability of the method for resolving potential interfering compounds.

In order to develop a method with the shortest possible run time, the aim was to use the isocratic reversed phase mode rather than the gradient mode. The HPLC–MS/MS method which employed a C18 column in isocratic mode eliminated the need for column equilibration in each run resulting in a fast analytical run. Method optimisation achieved a sharp peak for the folic acid peak with short retention time (2.6 min) and a total run time of 3.5 min, as can be seen in Figure 3c.

#### 3.1.3. Sample Preparation

For sample preparation, direct precipitation of proteins using solvents such as methanol or acetonitrile is the simplest, most convenient, and cost-effective way in comparison with other methods such as liquid–liquid extraction and solid-phase extraction. An attempt to use methanol did not yield good results due to poor chromatography and low peaks’ intensity. Better results were obtained when acetonitrile was used for precipitation. About 1.5 mL of cold acetonitrile was added to 0.5 mL of plasma and then 300 µL of the supernatant was evaporated to dryness and reconstituted in 200 µL of the mobile phase. Although higher intensities were achieved compared to methanol precipitation, intensity of LLOQ sample was very low. Consequently, the volume of the supernatant withdrawn was increased to 800 µL. The increase in volume improved signal intensities, but less clean samples were obtained and resulted in the formation of a brown to yellow layer on the electrospray ionisation chamber. A recentrifugation step was therefore introduced and applied to about 1 mL of the supernatant, and then 800 µL was withdrawn, evaporated to dryness, and reconstituted in 200 µL of mobile phase. This step gave cleaner samples, as deposition of matrix on the ionisation source was significantly reduced and higher intensities were achieved.

The developed method was superior to existing methods in terms of throughput: (i) the chromatographic analysis time is the shortest among existing methods [40,42,45,46]; (ii) and a simple sample preparation step of protein precipitation was utilised for preparation of plasma samples for folic acid analysis [41,42,45,46]. Additionally, deuterated I.S. was used in the present study, while most studies used other compounds that are structurally related to folic acid as I.S.s [40,45].

### 3.2. Method Validation

To test the linearity of the calibration curve of folic acid under the optimised conditions, nine calibration curves with eight nonzero concentrations (13.17 ng/mL, 26.33 ng/mL, 131.7 ng/mL, 439.9 ng/mL, 1024 ng/mL, 2194 ng/mL, 3292 ng/mL, and 3657 ng/mL) were prepared and injected. Excellent linearity was obtained between folic acid concentration and peak-area ratios of folic acid and folic acid-d4 over the tested concentration range (*r*^2^ > 0.995) as presented in Table 1. The LLOQ of the method was established at 13.17 ng/mL using 20 µL injection volume and 200 µL reconstitution volume. Although this injection volume produced sufficient sensitivity to quantify *in vivo* levels of folic acid in the bioavailability study, the LLOQ and sensitivity may be further improved by performing optimisation studies related to injection volume.

Within-run and between-run accuracy and precision data are presented in Table 2. The method was found to be accurate and precise. Within-run precision (CV%) ranged from 2.20% to 19.79%, while the accuracy percentage ranged from 90.94% to 104.36%. Similarly, interbatch precision percentage ranged from 3.14% to 13.15%, while the accuracy percentage ranged from 90.26% to 104.67%.

The reproducibility of the method was investigated and the method proved to be reproducible with a CV% values between 2.1% and 9.92% (Table 3).

The extraction recovery of folic acid and folic acid-d4 from human plasma was studied at four concentration levels—QCL, QCM-1, QCM-2, and QCH—each run in six replicates. The mean extraction recovery ranged from 79.16% to 92.95%, while the mean extraction recovery of folic acid-d4 was 115.86% (Table 4).

The matrix effect of the method was evaluated by analysing six lots of blank plasma spiked with folic acid at QCL and QCH concentrations after extraction, in comparison to pure solutions of folic acid. The I.S.-normalised MF and the corresponding CV% at QCL was found to be 103.00% and 2.47%, respectively, while for that at QCH was found to be 98.23% and 1.25%, respectively.

Short-term and long-term stability of stock solutions were evaluated and found to be stable for up to 48 h and 8 days, respectively. The stability of spiked QCL and QCH samples in plasma was assessed after storage at −20 °C and −70 °C for 9 days, after four freeze and thaw cycles at −20 °C and −70 °C, autosampler stability at room temperature and at 4 °C, dry extract stability at room temperature and at −20 °C, benchtop stability at room temperature, and stability in blood at room temperature. The stability results presented in Table 5 indicate that the folic acid in the tested samples was stable under the different investigated conditions.

Small interconversion THF and DHF to folic acid during sample preparation is possible, but not the other way around. THF and DHF, however, were not spiked into the samples in this study. For the real samples acquired from subjects, if endogenous folate, which normally exists in high concentration in plasma, had converted to folic acid during sample preparation, folic acid peaks would be detected in the predosing samples (folate baseline). Folic acid was not detected in any of these samples. The simple and quick sample preparation and short analysis time of the reported method is also advantageous for preventing such conversion.

Dilution integrity of the method was evaluated by analysing six replicate samples that were prepared as spiked standards at two concentration levels, 7315 ng/mL and 10,972 ng/mL, representing approximately 2× and 3× the concentration of the ULOQ (3657 ng/mL) after 2- and 3-fold dilution, respectively. The precision (CV%) was found to be 5.97% and 2.29%, respectively. Bias percentage was found to be 14.8% and 7.7%, respectively.

Carry over was assessed by injecting a blank sample after a high concentration sample (ULOQ 3657 ng/mL), followed by injecting LLOQ (13.17 ng/mL) to assess blank/LLOQ ratio. There was no apparent carry over.

Multiple reaction monitoring chromatograms of blank plasma samples and a plasma sample at the LLOQ level demonstrated the selectivity of the method. No interfering components were observed in the blank samples. Moreover, no interfering peaks were detected from six commonly used medications as demonstrated by the analysis folic acid QCL samples spiked with these drugs.

**Figure 4:**
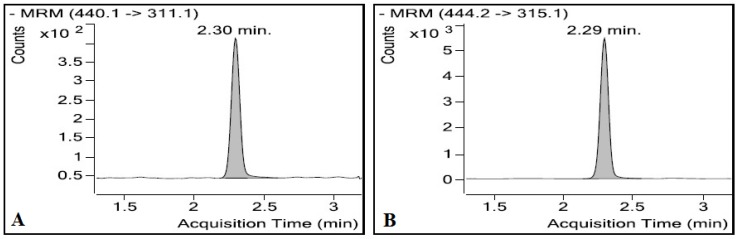
Representative chromatograms of a plasma sample (t = 5 h) from a healthy subject in a bioavailability study showing (**A**) folic acid and (**B**) I.S.

The developed method was applied successfully for the determination of folic acid levels in human plasma samples in a bioavailability study after the oral administration of a single folic acid dose (5 mg). Figure 4 is a representative chromatogram of folic acid and I.S. obtained for the analysis of a plasma sample (t = 5 h) obtained from one subject. This is a typical chromatogram used for calculation of folic acid concentration in the study samples.

**Figure 5:**
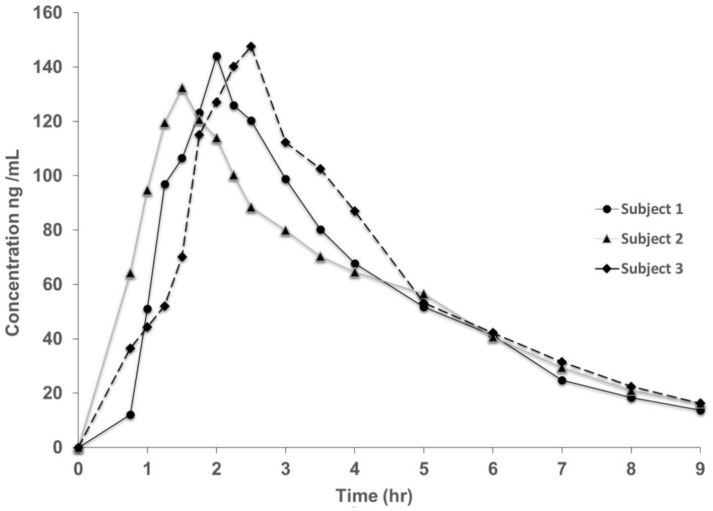
Plasma concentration–time curves of folic acid after oral administration of 5 mg folic acid to three healthy subjects.

Plasma concentration–time profiles of folic acid for three healthy subjects are shown in Figure 5. The profiles show similar patterns and comparable pharmacokinetics parameters for folic acid. The peak time of folic acid concentration (t_max_) was reached after 1.5–3 h with a mean 2 ± 0.5 h (mean ± standard deviation), whereas the maximum concentration (C_max_) ranged between 130–150 ng/mL with a mean 141 ± 8 ng/mL. Obtaining this data from subject samples demonstrates the applicability of the method for quantifying folic acid in real settings and its suitability to be used for other relevant applications such as clinical and epidemiological studies.

## 4. Conclusions

The health concerns associated with unmetabolised folic acid in human plasma in the population require reliable and high-throughput quantification methods to establish its levels *in vivo* and to investigate any potential links with diseases. A sensitive, accurate, rapid, and reproducible LC–MS/MS method was developed and validated for quantification of unmetabolised folic acid in human plasma in the range 13.17–3657 ng/mL. The method proved to be accurate and precise for the tested validation parameters. The short run time and minimal sample preparation make it a method of choice for bioavailability and epidemiological studies for folic acid. The method was applied successfully for the determination of unmetabolised folic acid levels in human plasma *in vivo* following administration of 5 mg tablet. Higher sensitivity and lower LLOQ, however, might be required for quantifying lower levels of folic acid levels. Populations who are not taking folic acid supplements but are obtaining folate from a normal diet enriched with folic acid are expected to produce much lower levels of folic acid in their plasma. Further studies related to improving ionisation efficiency and increasing sensitivity should be considered in future work particularly when considering population groups that are not using additional sources of folic acid.

## Figures and Tables

**Table 1:** Calibration curve parameters (*n* = 9).

Parameters	Linear Range (ng/mL)	Slope Mean ± SD	Intercept Mean ± SD	Correlation Coefficient Mean ± SD
Plasma sample	13.17–3657	0.00044 ± 0.00001	−0.0009 ± 0.0006	0.995 ± 0.004

**Table 2:** Intra- and interday precision and accuracy of the method for determination of folic acid in human plasma.

QC Level	Nominal Conc. (ng/mL)	Intrabatch (*n* = 6; Single Batch)			Interbatch (*n* = 30; 6 From Each Batch)		
		Measured Mean Conc. (ng/mL)	CV%	Accuracy%	Measured Mean Conc. (ng/mL)	CV%	Accuracy%
LLOQ QC	13.17	12.49	19.79	94.88	12.88	13.15	97.84
QCL	39.50	39.83	13.78	100.83	38.30	11.00	96.97
QCM-1	731.5	665.2	2.58	90.94	660.3	3.56	90.26
QCM-2	1829	1800	3.96	98.41	1813.	3.89	99.14
QCH	2743	2863	2.20	104.36	2871	3.14	104.67

QC: quality control; n: number of replicates; CV: coefficient of variation; LLOQ QC: lower limit of quantitation quality control; QCL: quality control low; QCM: quality control medium; QCH: quality control high.

**Table 3:** Reproducibility of the method for determination of folic acid in human plasma.

QC Level	Nominal Conc. (ng/mL)	Measured Mean Conc. (ng/mL) (*n* = 6)	CV%	Accuracy%
LLOQ QC	13.17	12.62	9.92	95.82
QCL	39.51	35.72	7.05	90.43
QCM-1	731.5	655.9	2.45	89.67
QCM-2	1829	1838	4.15	100.50
QCH	2743	2950	2.10	107.56

QC: quality control; n: number of replicates; CV: coefficient of variation; LLOQ QC: lower limit of quantitation quality control; QCL: quality control low; QCM: quality control medium; QCH: quality control high.

**Table 4:** Extraction recovery for folic acid.

QC Level	Area Response (Replicate, *n* = 6)		Mean Recovery %
	Extracted Sample Analyte Response Ratio	Untreated Standard Analyte Response Ratio	
QCL	0.0145	0.0156	92.95
QCM-1	0.2775	0.3408	81.43
QCM-2	0.7504	0.9479	79.16
QCH	1.2797	1.4823	86.33
I.S.	0.7818	0.6748	115.86

QC: quality control; n: number of replicates; QCL: quality control low; QCM: quality control medium; QCH: quality control high; I.S.: internal standard.

**Table 5:** Stability of folic acid QCL and QCH samples in plasma (*n* = 6).

Stability Test	Nominal Conc. (ng/mL) ± SD	Mean Stability Sample Conc. (ng/mL) ± SD	Change %
Stability in blood at room temperature for 1 h	51.8 ± 0.8	49.4 ± 3.5	−4.69
	3934 ± 78	3815 ± 30	−3.01
Long term stability at −20 °C for 9 day	35.6 ± 3.4	36.3 ± 1.6	1.94
	2757 ± 57	2778.864 ± 38	0.79
Long term stability at −70 °C for 9 day	35.6 ± 3.4	37.8 ± 2.1	5.99
	2757 ± 57	2761 ± 80	0.14
Freeze-thaw stability at −20 °C	33.8 ± 2.5	36.7 ± 2.3	8.72
	2866 ± 81	2830 ± 45	−1.26
Freeze-thaw stability at −70 °C	33.8 ± 2.5	37.9 ± 2.4	12.19
	2866 ± 81	2872 ± 91	0.20
Autosampler stability at room temperature for 123 h	39.8 ± 3.7	40.9 ± 5.5	2.90
	2862 ± 73	2864 ± 109	0.09
Autosampler stability at 4 °C for 124 h	39.7 ± 3.6	35.9 ± 3.0	−9.6
	2862 ± 73	2890 ± 93	0.97
Dry extract stability at room temperature for 125 h	39.7 ± 3.6	37.9 ± 3.6	−4.71
	2862 ± 73	2711 ± 69	−5.27
Dry extract stability at −20 °C for 120 h	39.7 ± 3.6	38.4 ± 4.0	−3.47
	2862 ± 73	2787 ± 49	−2.63
Bench-top stability at room temperature for 24 h	33.8 ± 2.5	34.1 ± 4.0	1.01
	2866 ± 81	2905 ± 64	1.35

## References

[1] Cahill L.E., El-Sohemy A., Erdman J.W., MacDonald I.A., Zeisel S.H. (2012). Genetic variation and nutrient metabolism: Folate. Present Knowledge in Nutrition.

[2] Nollet L.M.L., Toldra F. (2012). Food Analysis by HPLC.

[3] Bailey L.B., da Silva V., West A.A., Caudill M.A., Zempleni J., Suttie J.W., Gregory J.F., Stover P.J. (2013). Folate. Handbook of Vitamins.

[4] Institute of Medicine (1998). Dietary Reference Intakes for Thiamin, Riboflavin, Niacin, Vitamin B6, Folate, Vitamin B12, Pantothenic Acid, Biotin, and Choline.

[5] Pietrzik K., Bailey L., Shane B. (2010). Folic acid and L-5-methyltetrahydrofolate. Clin. Pharmacokinet..

[6] Rima O., Wolfgang H. (2012). The Emerging Role of Unmetabolized Folic Acid in Human Diseases: Myth or Reality?. Curr. Drug Metab..

[7] Stabler S.P., Bailey L.B. (2009). Clinical folate deficiency. Folate in Health and Disease.

[8] Hesdorffer C.S., Longo D.L. (2015). Drug-induced megaloblastic anemia. N. Engl. J. Med..

[9] Kalin S.R., Rimm E.B., Bailey L.B. (2009). Folate and vascular disease: Epidemiological perspective. Folate in Health and Disease.

[10] Zeng R., Xu C.-H., Xu Y.-N., Wang Y.-L., Wang M. (2015). The effect of folate fortification on folic acid-based homocysteine-lowering intervention and stroke risk: A meta-analysis. Public Health Nutr..

[11] Keum N., Giovannucci E.L. (2014). Folic acid fortification and colorectal cancer risk. Am. J. Prev. Med..

[12] Chen J., Xu X., Liu A., Ulrich C.M., Bailey L.B. (2009). Folate and cancer: Epidemiological perspective. Folate in Health and Disease.

[13] Araújo J.R., Martel F., Borges N., Araújo J.M., Keating E. (2015). Folates and aging: Role in mild cognitive impairment, dementia and depression. Ageing Res. Rev..

[14] Morris M.S., Jacques P.F., Bailey L.B. (2009). Folate and neurological function: Epidemiological perspective. Folate in Health and Disease.

[15] Tamura T., Picciano M.F., McGuire M.K., Bailey L.B. (2009). Folate in pregnancy and lactation. Folate in Health and Disease.

[16] Scholl T.O., Johnson W.G. (2000). Folic acid: Influence on the outcome of pregnancy. Am. J. Clin. Nutr..

[17] Huhta J.C., Linask K. (2015). When should we prescribe high-dose folic acid to prevent congenital heart defects?. Curr. Opin. Cardiol..

[18] Greene N.D.E., Copp A.J. (2014). Neural tube defects. Annu. Rev. Neurosci..

[19] Hobbs C.A., Shaw G.M., Werler M.M., Mosley B., Bailey L.B. (2009). Folate status and birth defect risk: Epidemiological perspective. Folate in Health and Disease.

[20] De-Regil L.M., Peña-Rosas J.P., Fernández-Gaxiola A.C., Rayco-Solon P. (2015). Effects and safety of periconceptional oral folate supplementation for preventing birth defects. Cochrane Database Syst. Rev..

[21] Castillo-Lancellotti C., Tur J.A., Uauy R. (2013). Impact of folic acid fortification of flour on neural tube defects: A systematic review. Public Health Nutr..

[22] Bailey S.W., Ayling J.E. (2009). The extremely slow and variable activity of dihydrofolate reductase in human liver and its implications for high folic acid intake. Proc. Natl. Acad. Sci. USA.

[23] Wright A.J.A., Dainty J.R., Finglas P.M. (2007). Folic acid metabolism in human subjects revisited: Potential implications for proposed mandatory folic acid fortification in the UK. Br. J. Nutr..

[24] Mason J.B., Dickstein A., Jacques P.F., Haggarty P., Selhub J., Dallal G., Rosenberg I.H. (2007). A temporal association between folic acid fortification and an increase in colorectal cancer rates may be illuminating important biological principles: A hypothesis. Cancer Epidemiol. Biomarkers Prev..

[25] Kim Y.-I. (2007). Folate and colorectal cancer: An evidence-based critical review. Mol. Nutr. Food Res..

[26] Williams E.A. (2012). Folate, colorectal cancer and the involvement of DNA methylation. Proc. Nutr. Soc..

[27] Mudryj A.N., de Groh M., Aukema H.M., Yu N. (2016). Folate intakes from diet and supplements may place certain Canadians at risk for folic acid toxicity. Br. J. Nutr..

[28] Cuskelly G.J., Mooney K.M., Young I.S. (2007). Folate and vitamin B12: Friendly or enemy nutrients for the elderly: Symposium on ‘Micronutrients through the life cycle’. Proc. Nutr. Soc..

[29] Selhub J., Rosenberg I.H. (2016). Excessive folic acid intake and relation to adverse health outcome. Biochimie.

[30] Kim Y.I. (2006). Folate: A magic bullet or a double edged sword for colorectal cancer prevention?. Gut.

[31] Mason J.B. (2011). Unraveling the complex relationship between folate and cancer risk. BioFactors.

[32] Figueiredo J.C., Grau M.V., Haile R.W., Sandler R.S., Summers R.W., Bresalier R.S., Burke C.A., McKeown-Eyssen G.E., Baron J.A. (2009). Folic acid and risk of prostate cancer: Results from a randomized clinical trial. JNCI, J. Natl. Cancer Inst..

[33] O'Broin S., Kelleher B. (1992). Microbiological assay on microtitre plates of folate in serum and red cells. J. Clin. Pathol..

[34] Iyer R., Tomar S.K. (2013). Determination of folate/folic acid level in milk by microbiological assay, immuno assay and high performance liquid chromatography. J. Dairy Res..

[35] Pfeiffer C.M., Fazili Z., Zhang M., Bailey L.B. (2009). Folate analytical methodology. Folate in Health and Disease.

[36] Osseyi E.S., Wehling R.L., Albrecht J.A. (1998). Liquid chromatographic method for determining added folic acid in fortified cereal products1. J. Chromatogr. A.

[37] Ichinose N., Tsuneyoshi T., Kato M., Suzuki T., Ikeda S. (1993). Fluorescent high-performance liquid chromatography of folic acid and its derivatives using permanganate as a fluorogenic reagent. Fresenius J. Anal. Chem..

[38] Kalmbach R., Paul L., Selhub J. (2011). Determination of unmetabolized folic acid in human plasma using affinity HPLC. Am. J. Clin. Nutr..

[39] Pawlosky R.J., Flanagan V.P. (2001). A quantitative stable-isotope lC−MS method for the determination of folic acid in fortified foods. J. Agric. Food Chem..

[40] Zheng X.-H., Jiang L.-Y., Zhao L.-T., Zhang Q.-Y., Ding L. (2015). Simultaneous quantitation of folic acid and 5-methyltetrahydrofolic acid in human plasma by HPLC–MS/MS and its application to a pharmacokinetic study. J. Pharm. Anal..

[41] Kirsch S.H., Knapp J.-P., Herrmann W., Obeid R. (2010). Quantification of key folate forms in serum using stable-isotope dilution ultra performance liquid chromatography–tandem mass spectrometry. J. Chromatogr. B.

[42] Fazili Z., Whitehead R.D., Paladugula N., Pfeiffer C.M. (2013). A high-throughput LC-MS/MS method suitable for population biomonitoring measures five serum folate vitamers and one oxidation product. Anal. Bioanal. Chem..

[43] Garbis S.D., Melse-Boonstra A., West C.E., van Breemen R.B. (2001). Determination of folates in human plasma using hydrophilic interaction chromatography−tandem mass spectrometry. Anal. Chem..

[44] FDA Guidance (2013). Guidance for Industry. Bioanalytical Method Validation. https://www.fda.gov/downloads/Drugs/guidancecomplianceregulatoryinformation/guidances/ucm368107.pdf.

[45] Álvarez-Sánchez B., Priego-Capote F., Mata-Granados J.M., Luque de Castro M.D. (2010). Automated determination of folate catabolites in human biofluids (urine, breast milk and serum) by on-line SPE–HILIC–MS/MS. J. Chromatogr. A.

[46] Rychlik M., Netzel M., Pfannebecker I., Frank T., Bitsch I. (2003). Application of stable isotope dilution assays based on liquid chromatography–tandem mass spectrometry for the assessment of folate bioavailability. J. Chromatogr. B.

